# Coping during COVID-19 Pandemic in Saudi Community: Religious Attitudes, Practices and Associated Factors

**DOI:** 10.3390/ijerph18168651

**Published:** 2021-08-16

**Authors:** Fahad D. Algahtani, Mohamed Ali Alzain, Najoua Haouas, Khadijah Angawi, Bandar Alsaif, Adel Kadri, Mohamed A. Dkhil, Mejdi Snoussi, Rafat Zrieq

**Affiliations:** 1 Department of Public Health, College of Public Health and Health Informatics, University of Ha’il, Ha’il 55476, Saudi Arabia; f.algahtani@uoh.edu.sa (F.D.A.); m.alzain@uoh.edu.sa (M.A.A.); b.alsaif@uoh.edu.sa (B.A.); 2 Molecular Diagnostic and Personalised Therapeutics Unit, University of Ha’il, Ha’il 55476, Saudi Arabia; 3 Laboratory of Medical and Molecular Parasitology-Mycology LP3M (Code LR12ES08), Department of Clinical Biology, Faculty of Pharmacy, University of Monastir, Monastir 5000, Tunisia; najoua.h@laposte.net; 4 Department of Health Services and Hospital Administration, Faculty of Economics and Administration, King Abdulaziz University, Jeddah 80200, Saudi Arabia; kkangawi@kau.edu.sa; 5 College of Science and Arts in Baljurashi, Albaha University, Albaha 1988, Saudi Arabia; lukadel@yahoo.fr; 6 Department of Zoology, College of Science, King Saud University, Riyadh 11795, Saudi Arabia; mohameddkhil@yahoo.com; 7 Department of Biology, College of Science, University of Ha’il, Ha’il 55476, Saudi Arabia; snmejdi@yahoo.fr

**Keywords:** COVID-19, pandemic, religion, Islam, prevention measures, health education intervention, KSA

## Abstract

The COVID-19 pandemic has affected many societies’ life aspects and activities including social and Islamic practices; more attention should be given to investigate the interaction between Islamic worships and the spread of the disease. Here, we performed a cross-sectional study using an online questionnaire to assess the preventive Islamic attitudes and practices during the COVID-19 lockdown period from the Saudi publics’ perspectives. Mann-Whitney, Kruskal and logistic regression tests were used to analyze the data. The results revealed that most participants had positive Islamic attitudes and practices. However, elders and males were less obeyed to preventive measures during performing worship (*p* < 0.05). While younger, females and not married were less obeyed when dealing with COVID-19 related death (*p* < 0.05). Even though, elders were less likely to have poor social and Islamic practices concerning adherence to preventive measures during the pandemic (OR = 0.38; 95% CI: 0.176–0.816) than younger. Furthermore, males, Saudi participants, lower education level, lower Islamic attitudes scores were more likely to have poor social and Islamic practices concerning adherence to preventive measures during the pandemic (OR = 1.65; 95% CI: 1.126–2.421; OR = 1.57; 95% CI: 1.067–2.322; OR = 3.09; 95% CI: 1.721–5.563; and OR = 1.89; 95% CI: 1.309–2.753, respectively), than their corresponding targeted counterparts. Thus, despite the high preventative perceptions of Islamic attitudes and practices of the Saudi community, our study highlighted some risk groups with less preventative practices. Thus, targeted health education interventions are highly recommended for these risk groups to enhance the commitment to government instructions.

## 1. Introduction

The World Health Organization (WHO) declared COVID-19 as a global pandemic emergency due to its vast spread across many different countries, affecting all aspects of life including social, political and religious activities among societies [1]. In the absence of any effective treatment, albeit the arising vaccines and the big debate about it, countries have undergone social behavioral changes including lockdowns and social distancing measures to curb the COVID-19 transmission rate [2,3].

However, increasing attention is being paid to investigate the role of religious practices changes as well as beliefs and the impact of religious practices and perceptions on the COVID-19 transmission in the literatures. In this context, Saban and colleagues (2020) reported a huge variation in the incident rate of COVID-19 among the followers of different religions/denominations in the same community, who are subject to the same governmental measures. Such study suggested that religious practices and perceptions have likely pivotal roles in the spreading of the disease [4]. In fact, there are many religious practices and perceptions that can play a role as risk factors in the spread of COVID-19 such as pilgrimage, attending group praying and religious lessons in worship places, group dining tables associated with religious occasions, celebrating religious marriage ceremony as well as religious holidays and children circumcision… etc. Subsequently, these practices may result in the formation of infection foci. Thus, religious leaderships, by adapting alterations in the religious rituals and performance of certain practices, play a pivotal role in combating the spread of disease, either positively or negatively [5]. For instance, Iran was one of the most affected countries in the Islamic region because leaders were encouraging the religious ceremonies and gatherings that impacted the fast spread of the disease. On 19 February 2020, the first cases of COVID-related deaths occurred at the Shiite holy city of Qom as a result of encouraging people to perform the Shrines pilgrimage [6]. Other religious-related COVID-19 foci such Malaysia, Pakistan and India were reported due to the encouragement of Islam gatherings by spiritual leaders that led to the spread of the disease [7]. At the beginning of the outbreak in March 2020, South Korea was one example where 5000 cases were infected with COVID-19. These cases were infected from one case “Patient 31”, who prayed at Shincheonji Church of Jesus in Daegu [8,9]. The Christian leadership of that church insisted on the prayer gatherings and banned the wearing of face masks and refused to comply with government and public health officials’ instructions. Similarly, the Jewish ultra-Orthodox community, who committed with their religious leaders in ignoring the preventative measures as they interfere with their religious instructions, recorded a high COVID-19 incidence and death rate, twice as high in the general population [4]. In contrast, a Jerusalem synagogue adopted a strategy to cope with the coronavirus pandemic by serving its congregants and maintain community via streaming virtual religious lessons and organizing virtual prayer services [5]. Such practice has effectively reduced the incidence of COVID-19 infection among its congregants in comparison to their counterparts going to another synagogue.

Kingdom of Saudi Arabia (KSA), which is considered the most representing country for Islamic believes and practices as well as a hotspot for the biggest Islam mass gatherings worldwide during Hajj (the greater Islamic pilgrimage) and Umrah (the small Islamic pilgrimage), host over three million pilgrims yearly at the same time [3]. In these gatherings, people come together within close proximity. Since Islamic gatherings and events were considered as threats when mitigating the spread of an epidemic, KSA public health authorities and government officials have promptly and effectively responded to the outbreak crises of COVID-19 at an early stage. Nevertheless, common Muslim religious practices—such as gathering and praying in the mosque for praying 5 times a day, Friday prayer (usually the most crowded prayer of the week as it is obligatory to be conducted in the mosque), attendance of Islamic instructional lessons, ritual washing and kissing of the deceased, joining in the funeral and mourners gathering for few days at the deceased family house could have undermined the governmental efforts and facilitate the spread of infectious diseases including COVID-19 [10]. Therefore, the Saudi authorities have imposed unprecedented precautionary strict measures to the community including lockdown, quarantine, isolation and social distancing to reduce the incidence rate of the pandemic. These measures were immediately implemented with the recording of the first case in the country on 2 March 2020 [11]. Additionally, tourist visas, international and domestic flights, schools and malls were suspended to halt the spread of the diseases. At the religion level, government banned Hajj and Umrah visas, activities held in mosques and entry to all mosques in the country including the Two Holy Mosques in Mecca and Medina. These efforts were undertaken in collaboration with the Islamic leaders in the country.

Unfortunately, there is no available tool to measure the impact of applying precautionary measures in the light of developing religious verdicts and the adaptation of religious practices on limiting the spread of disease in any community due to the difficulty of separating the religion factors from other influencing factors. Instead, religious attitudes and practices during the pandemic were used as indicators for the influence of conducting religious practices on the spread of the disease. Therefore, this study aimed to assess the Islamic practices and believes on the spread and prevention of the COVID-19 pandemic from the Saudi publics’ perspectives, which will help understanding the social and Islamic expectations that could prevent the spread of COVID-19. Results from this study could be vital for the development and design of effective health interventions during the COVID-19 pandemic.

## 2. Materials and Methods

### 2.1. Study Design, Procedure and Participants

An online cross-section survey was conducted between 15 July to 5 October 2020, among adult’s population aged ≥18 years in the KSA. The online survey was the favorite method due to the precaution measures implemented during the pandemic. The survey was designed using Google Form and generating link. The link was distributed through social media (Twitter and WhatsApp) to eligible participants using a convenient sampling method.

### 2.2. Sample Size

The minimum sample size was (385) when calculated by the following formula: $\frac{z^{2}P\left(1-P\right)}{d^{2}}$ [12].

Whereas the assumption of the proportion of good Islamic attitude and practice toward COVID-19 is 50%, with a 95% confidence interval and 5% marginal error. A total of 464 completed questionnaires were received and included in the study.

### 2.3. Data Collection

The survey contained two sections: first demographic characteristics (gender, age, occupation, marital status and educational level); second, assessment of the Islamic attitudes and practices toward prevention of COVID-19. The Islamic attitudes was defined as the way the population thinks and behaves about suspending Umrah, how to perform rituals in the mosque and dealing with sick and dead from COVID-19. It was measured by 10 questions with a five-point Likert’s scale; strongly agree, agree, somehow, disagree, strongly disagree (5–1 points), respectively. The practice was defined as the habit of the population toward performing some social and Islamic activities, e.g., regular prayers in the Mosque and practicing social distance measures. The practice section had nine items, and each item was answered by ‘always’ (1 point), ‘sometimes’ (2 point) or ‘never’ (3 point). To improve the quality of data collection, a pretested was performed to measure the reliability of Islamic attitudes and practices using Cronbach’s alpha test (0.85 and 0.74), respectively. To ensure the validity of the questionnaire, specialists in the public health field, Islamic leaders and statisticians reviewed the content and construct of the questionnaire to meet the study’s objectives.

### 2.4. Data Management and Analysis

The data were analyzed using SPSS version 25 (IBM Corporation, Armonk, NY, USA). Frequencies and percentages were used to represent the demographic variables, and different participants’ responses for Islamic attitudes and practices. Shapiro-wilk test was performed to examine the normality of continuous variables. Median and interquartile range were used since data were non-normally distributed. The total scores of Islamic attitudes were computed resulting in a median of 42 (Interquartile Range (IQR): 38–46) and ranging between 10 and 50; whereas the median score of social and Islamic practice was 23 (IQR: 21–25) ranging from 10 to 27. Mann-Whitney and Kruskal Wallis tests were used to identify the median difference between groups. Logistic regressions test was performed to predict lower social and Islamic practice (score < median) using attitude and significant demographic.

A principal component analysis (PCA) was performed on attitude questions. The suitability of PCA was assessed through correlation matrix, Kaiser-Meyer-Olkin and Bartlett’s tests. All results were two-sided, and *p* value less than 0.05 was considered significant.

## 3. Results

This section may be divided by subheadings. It should provide a concise and precise description of the experimental results, their interpretation, as well as the experimental conclusions that can be drawn.

### 3.1. Demographic Characteristics

A total of 464 participants completed the online survey. About 61% were males, and 41.4% were in age group between 20–30 years. The majority (63.8%) of participants were citizens, and more than half (50.6%), (52.4%) and (53.9%) had bachelor’s degree, employed and married, respectively, as shown in Table 1.

### 3.2. Islamic Attitudes

Table 2 reveals the proportion of answers on the five-point Likert scale on the different Islamic attitudes toward COVID-19 prevention items. Most respondents had higher attitude scores (responses ranged from agree to strongly agree) towards different measures taking by the KSA government. The participants strongly agreed (Median = 5) on the suspension of Umrah in Mecca, preventing the elderly, chronic diseases patients and children from praying in the mosque. As well as social distancing, wearing masks and shorten the period of staying in the mosque before and after prayer as efficient preventing measures to reduce the possibility of COVID-19 transfer among the Saudi community. To less extent, the participants’ perception toward wearing the Niqab (a face cover used by some Muslim women), prohibit holding Quran memorization and Islamic lessons in mosques and prohibiting hand over, last look and kissing of COVID-19 related-dead, received relatively high attention (Median = 4) of the participants. These results imply that the attitude of the Saudi community is in high agreement with the prevention measures taken by their government and the flexibility in Fataws (formal legal opinion issued by Islamic leaders according to the Islamic rules) to encounter the spread of the disease.

### 3.3. Social and Islamic Practices

Table 3 shows the proportion of responses on a three-point Likert scale on the various social and Islamic practices towards COVID-19 prevention items. Almost all practices received high positive practices regarding the prevention measures (Median = 3). Most of the respondents avoid visiting the elderly, shaking hands, kissing and going to the crowded place 55%, 54.5%, 57.1% and 66.4%, respectively. While most of them never perform Friday congregational prayers in the Mosque, gathering family, visiting mourning place, social events and visiting patients in the hospital, 39.7%, 60.1%, 76.3%, 68.3% and 42%, respectively. These results indicate that the Saudi community strongly supported and practiced the prevention measures concerning both Islamic practices and, albeit to less extent, social habits.

### 3.4. Impact of Demographic Factors on the Scores of Islamic Attitudes and Practices

Table 4 shows the effect of demographic factors on the scores of Islamic attitudes and practices. We observed no significant differences among the demographic factors tested and participants’ attitude. Interestingly, the results showed that housewives and jobseekers had significantly higher Islamic attitude scores than other occupations.

On the other hand, all demographic factors tested showed significant differences on the social and Islamic practice scores (*p* value < 0.02). Furthermore, the median of social and Islamic practices was significantly higher among the participants >30 years old than younger participants (≤30 years), with the lowest median observed in <20. Furthermore, females, residents, postgraduates, as well as married and divorced participants had significantly higher social and Islamic practice scores than their corresponding targeted counterparts. Additionally, employed and Job-seekers participants had significantly higher social and Islamic practice scores than other occupations tested. Collectively, the results showed that the age and occupation variables had significant differences in social and Islamic attitudes while all variables had significant differences in social and Islamic practices.

The logistic regression was performed to predict lower social and Islamic practice (score < median) using significant demographic factors and Islamic attitude scores. The results revealed that elder participants (>30 years) were less likely to have lower social and Islamic practice score (OR = 0.38; 95% CI: 0.176–0.816; OR = 0.23; 95% CI: 0.101–0.509; and OR = 0.38; 95% CI: 0.144–1.026), respectively, than younger participants (<20 years). Furthermore, males were more than one and a half time (OR = 1.65; 95% CI: 1.126–2.421) more likely to have lower social and Islamic practice scores than females. Saudi participants were more than one and half (OR = 1.57; 95% CI: 1.067–2.322) times more likely to have lower social and Islamic practice scores than non-Saudi. Furthermore, the education less or at university level have lower social and Islamic practice scores compared to post-graduate (OR = 3.09; 95% CI: 1.721–5.563) and (OR = 1.70; 95% CI: 1.122–2.585), respectively. Subsequently participants with lower Islamic attitude scores (<Median) had almost two times (OR = 1.89; 95% CI: 1.309–2.753) more likely to have lower social and Islamic practice scores than those with ≥Median Islamic attitude scores, as shown in Table 5.

To improve the strength of the Islamic attitude factors, principal component analysis was performed (Table 6). To do so, two components were extracted when the rotation converged in two iterations. The two components were performed worship (component 1: seven factors) and dealt with suspected or confirmed COVID-19 related deaths (component 2: two factors), which explain 58.6%, while KMO was 0.782 indicating a meritorious level, and Bartlett’s test for sphericity was significant (χ^2^ = 1518.279, *p* ≤ 0.001). The reliability of these components was 0.836 and 0.838, respectively. Thus, these tests demonstrated that sampling adequacy for each variable in the model and for the complete model is highly suited and our sample is taken from populations with equal variances.

After splitting the Islamic attitudes into two components (i.e., worship and ‘dealing with death’), the association between demographic factors and these components were analyzed. As shown in Table 7, the median scores of both Islamic attitude components were statistically significant among different age groups (*p* value < 0.05) and showed lower worship attitudes among elders (>50 years) than other age groups. In contrast, the median of younger was lower in the ‘dealing with death’ component (*p* value < 0.05). Additionally, the median of the worship component was significantly higher among females, housewives and job seekers (*p* value < 0.05) than their corresponding counterparts. On the other hand, the median of the ‘dealing with death’ component was significantly higher among male, residents, postgraduate and divorced participants than their corresponding counterparts (*p* value < 0.05). Furthermore, employed, job seekers and retired had significantly higher scores than other occupations (*p* value < 0.05). Collectively, younger, female, Saudi citizens, highly educated, divorced and housewives had higher attitude of worship during the pandemic. On the other hand, ‘dealing with death’ had higher attitude by elder, male, residents, highly educated, divorced, housewives and job seekers.

## 4. Discussion

COVID-19 pandemic is disseminating rapidly worldwide. This pandemic has very significant impact on the whole social activities including Islam life. Therefore, in this study, we investigated the Islamic attitudes and practices of the Saudi community during the lockdown periods in KSA. This study revealed that most participants had positive Islamic attitudes and practices toward different government prevention measures, as shown in Table 2, Table 3 and Table 4. The majority of participants were compliant with authorities in terms of avoidance of social and Islamic gatherings. More than two-thirds of the population supported the decision of Umrah and Hajj suspensions, as well as preventing the elderly and people with chronic diseases from praying in the mosque and strongly believed it was necessary to control the spread of the pandemic. Our results were consistent with the results of Alahdal et al. who reported a high attitude perception of the Saudi community (93% of the respondents) toward the ban of Umrah and Hajj as well as good practices (e.g., avoiding shaking hands, 86.9%) to limit the spread of COVID-19 [13].

Such public’s high agreement is derived from their confidence in the Saudi government decision to suspend all Islamic mass gathering due to rise in number cases of COVID-19 [14]. Such confidence is a direct result of the previous valuable experience of Saudi Health Authorities with the management of emerging infectious diseases either during the annual Hajj or unexpected outbreak such as MERS-CoV infection. Indeed, Saudi Health Authorities gained this trust from their experience in dealing with several emerging infectious diseases during annual Hajj seasons, such as Alkhumra virus infection and influenza A (H1N1) infection [15]. Same, since 2012, the tremendous experience learned by Saudi Arabia from dealing with MERS-CoV infection outbreak has allowed KSA to better respond to the current COVID-19 pandemic. This is also likely supported by the high confidence of the Saudi community (more than 90%) in the Saudi government’s control measures during the pandemic [16]. Consequently, majority of participants in the current study were compliant with authorities in terms of avoidance of social and Islamic gathering.

Additionally, this might be also due to the high awareness of preventive measures among the Saudi community which positively contributes to desirable attitudes and practices and improves infection prevention and control [17]. Moreover, as an Islamic country, Saudi community is deeply informed about Islamic rules and history which are rich in prevention measures and pandemic stories. As an example, it is stated in the Qur’an, Al Baqarah verse 195, “Do not throw yourselves with your own hands into destruction”. The Prophet Mohammad also emphasized commitment with distancing during pandemics, as stated in his speech “If you get wind of the outbreak of plague in a land, do not enter it; and if it breaks out in a land in which you are, do not leave it” [18]. Furthermore, in Islamic history, the idea of ‘quarantine’ or ‘social isolation’ as means to control contagion proposed by the father of early modern medicine Ibn Sina (Avicenna) is also well known to the Muslim communities. Our results also emphasize the positive response of the Saudi community to the Islamic leaders who have had a major role in the adaptation of Islamic practices to the pandemic conditions and subsequently influencing the public perception of virus containment [19]. In this context, our results might support the recently proposed collaborative model between Islamic communities and healthcare providers/policymakers to manage the COVID-19 and to engage the Islamic leaders of the respective societies and communities as frontline professionals [20,21].

Our current study reported a statistically significant association between positive worship attitudes towards COVID-19 pandemic and younger, females, job seekers and housewives, respectively. However, elders (>50 years) and males were less agreed on the Islamic restrictions being implemented in the worship to prevent the spread of the pandemic compared to other groups. This could be because age is adversely correlated with the level of understanding of COVID-19 [22]. Additionally, others’ studies reported housewives and job seekers significantly highly associated with the perspectives of Islamic attitudes [23]. Similarly, Indonesian females were also reported avoiding prayers, physical gatherings and take preventive measures at worship places during the COVID-19 pandemic than males [24]. In general, our results are also consistent with recent studies conducted in Saudi Arabia which reported that younger and women have higher optimistic attitudes towards the COVID-19 pandemic than men [13,14,25,26,27]. These results can be attributed to the increased usage of the social media channels by younger, females, job seekers as well as housewives, which can improve the Islamic knowledge and attitudes regarding the disease, compared to their counterparts.

With regards to Islamic attitudes in handling the body of the dead and post-mortem practices, almost half of the participants agreed on preventive practices being made for handling bodies when a person has died because of COVID-19. This is linked with a previous study that assessed Ebola knowledge, attitudes and practices in West Africa showing that almost 60% of the participants preferred safe burials of the bodies infected with Ebola [28]. Even though the latter study did not measure burial practices, it is vitally similar to the current findings. Connectedly, this is in alignment with the world health organization’s burial guidelines that suggest avoiding the touch or kissing of a deceased body infected with COVID-19 [29]. Conversely, some other Muslim-majority communities had relatively poor attitudes toward Islamic obligations and prayers that Muslims attend, in which almost half of the studied population insist on attending burial rites and Friday prayer despite lockdown measures [30]. These differences could be attributed to the variation in the levels of awareness in Islam as non-Arabic Muslims have less understanding of Islam due to language barriers. Contrastingly younger, female, less educated, not married and certain occupation were less agreed on the restrictions being made when dealing with COVID-19 related deaths compared to other groups.

Regarding social and Islamic practices during the COVID-19 pandemic, the majority of the participants incised the importance to avoid visiting family and friends, shaking hands and crowded places. This is consistent with other studies in Saudi Arabia [13,14,31]. Additionally, almost 40% avoided performing the usual and Friday congregational prayers in the mosque during the COVID-19 pandemic. Indeed, the adaptation of practices extended from Islamic rules to positively curb the impact of pandemics had also been shown for other Muslim-minority communities in other countries, which contrasted with the numerous reports and the hypothesis that ethnic minorities have poor health outcomes. For instance, in a multicultural and Islamic community, it has recently been reported that the COVID-19 infection rate in the general Jewish population was twice higher compared to the Arab (Muslim) population. Moreover, the Arab/Muslim mortality rate due to COVID-19 infection was 0.57 per 100,000, compared to 3.37 in the overall population, and to 7.26 in the ultra-Orthodox community. These variations were attributed to the difference in Islamic attitudes and practices [4]. Remarkably, our and other studies emphasized how Islam rules are highly fit to prevention measures during pandemics, in particular for the case of COVID-19.

In respect of social and Islamic practices, our study proved that younger (<20 years), males, residents, less educated and participants with lower Islamic attitudes score were significantly associated with poor social and Islamic practices compared to their counterparts. Our data are compatible with other studies which reported a positive significant association between Islamic practice and females, elders [14,24,31]. Moreover, our results also consistent with other studies which showed that females and highly educated are more committed to strong precautionary practices compared to their counterparts [32]. Our and others’ findings could be attributed to the participants’ (females and post-graduated) high knowledge of COVID-19 which would be consequently translated into optimistic practices during the COVID-19 pandemic and the low responsibility commitments of the other groups, which allowed them to more focus on positive Islamic practices in relation to preventive measures.

## 5. Conclusions

This study sheds light on the levels of attitudes and practices of intervention measures related to Islamic worship and rituals and reported a high response in the Saudi society in applying the precautionary measures in this regard. Our study highlights the importance of adherence to the application of Islamic practices in accordance with the rules issued by the government and the adaptation to engaging in worship according to the procedures issued by moderate clerics. There were certain socio-demographic factors closely related to perceptions of Islamic attitudes and practices during the COVID-19 pandemic in Saudi society, which highlighted some groups with practices that are less committed to precautionary measures, such as the younger, the less educated, unmarried and retired individuals. Our study recommends increasing the follow-up of these risk groups through targeted health education interventions.

This study has some limitations. There was no valid instrument to evaluate the perspectives of Islamic practices and restrictions in the literature and there is a dearth of empirical studies in this area. Another limitation was the small sample size that may affect the generalizability of the results to the population. The data was collected at the time during strict restrictions and closure of mosques due to the surge of COVID-19 cases. The risk perceptions of the public could have been impacted by these factors affecting the results of this study. The results could differ now with the social distancing restrictions have loosening and mosques are fully functioning with light restrictions. The strength of this study is that it is the first study evaluating the perspectives of Islamic practices and restrictions during COVID-19 among the Saudi population.

## Figures and Tables

**Table 1:** Demographic characteristics of the study participants.

Variables	Frequency	Percentage
Age Group		
<20 years	37	8%
20–30 years	192	41.4%
31–40 years	112	24.1%
41–50 years	92	19.8%
>50 years	31	6.4%
Gender		
Male	283	61%
Female	181	39%
Nationality		
Non-Saudi	168	36.2%
Saudi	296	63.8%
Education		
Less than university	58	14.6%
University (Bachelor)	235	50.6%
Post-graduate	161	34.7%
Marital status		
Not married	210	45.3%
Married	250	53.9%
Divorced	4	0.9%
Occupation		
Employee	243	52.4%
Freelance workers	8	1.7%
Students	9	1.9%
Housewives	186	40.1%
Job seekers	11	2.4%
Retired	7	1.5%

**Table 2:** The proportion of different Islamic attitudes toward COVID-19 among study population in KSA.

Question	S.A. *n* (%)	A. *n* (%)	S.H. *n* (%)	D. *n* (%)	S.D. *n* (%)	Median (IQR)	Result
Do you think the decision to suspend Umrah in Mecca is necessary to control the spread of COVID-19?	309 (66.6)	93 (20)	43 (9.3)	8 (1.7)	11 (2.4)	5 (4–5)	Strongly agree
Do you think preventing the elderly and people with chronic diseases from praying in the mosque is useful to minimize the spread of COVID-19?	247 (53.2)	113 (24.3)	75 (16.2)	25 (5.4)	4 (0.9)	5 (4–5)	Strongly agree
Do you think preventing children under 15 years from praying in the mosque is helpful to minimize the spread of COVID-19?	209 (45)	92 (19.8)	93 (20)	53 (11.4)	17 (3.6)	5 (4–5)	Strongly agree
Do you think imposing two meters-distance between worshipers while praying is useful to minimize the spread of COVID-19?	289 (62.3)	106 (22.9)	40 (8.6)	16 (3.4)	13 (2.8)	5 (4–5)	Strongly agree
Do you think wearing mask while praying in the mosque is necessary to control the spread of COVID-19?	314 (67.7)	87 (18.8)	38 (8.2)	15 (3.2)	10 (2.1)	5 (4–5)	Strongly agree
Do you think wearing the Niqab for a woman can reduce the risk of COVID-19 infection?	190 (40.9)	121 (26.1)	93 (20)	42 (9.1)	18 (3.9)	4 (4–5)	Agree
Do you think prohibiting hand-over of COVID-19 related-death can prevent minimize the spread of the epidemic?	147 (31.7)	108 (23.2)	89 (19.2)	83 (17.9)	37 (8)	4 (2–5)	Agree
Do you think prohibiting the last look and kissing of COVID-19 related-death can minimize the spread of the infection?	152 (32.7)	109 (23.5)	96 (20.7)	76 (16.4)	31 (6.7)	4 (3–5)	Agree
Do you think that shortening the time of staying in the mosque before and after prayer is helpful to minimize the spread of COVID-19?	287 (61.8)	104 (22.4)	43 (9.3)	24 (5.2)	6 (1.3)	5 (4–5)	Strongly agree
Do you think prohibiting the attendance of Islamic instruction courses in mosques is necessary to prevent the spread of the virus?	190 (40.9)	109 (23.5)	74 (16)	76 (16.4)	15 (3.2)	4 (3–5)	Agree

S.A.: Strongly agree; A.: Agree; S.H.: Somehow; D.: Disagree; S.D.: Strongly disagree IQR: Interquartile range.

**Table 3:** The proportion of different social and Islamic practices toward COVID-19 among study population in KSA.

Question	Always *n* (%)	Sometimes *n* (%)	Never *n* (%)	Median (IQR)	Result
Do you perform the 5-times-daily and Friday congregational prayers in the Mosque during the COVID-19 pandemic?	123 (26.6)	157 (33.8)	184 (39.7)	2 (1–3)	Sometimes
Do you avoid visiting relatives, in particular the elderly, to protect their health?	255 (55)	172 (37.1)	37 (8.0)	3 (2–3)	Always
Do you avoid shaking hand and keep almost two meters between you and others?	253 (54.5)	168 (36.2)	43 (9.3)	3 (2–3)	Always
Do you avoid going to crowded places unless it is necessary?	308 (66.4)	129 (27.8)	27 (5.8)	3 (2–3)	Always
Do you prefer gathering family and friends in the resort during the COVID-19 pandemic?	42 (9.1)	143 (30.8)	279 (60.1)	3 (2–3)	Never
Do you avoid kissing in greeting your relatives and friends?	265 (57.1)	141 (30.4)	58 (12.5)	3 (2–3)	Always
Are you interested in attending the condolence gathering places during the COVID-19 pandemic?	16 (3.4)	94 (20.3)	354 (76.3)	3 (3–3)	Never
Are you interested in attending social events (weddings, banquets, parties, etc.) during the COVID-19 pandemic?	18 (3.9)	129 (27.8)	317 (68.3)	3 (2–3)	Never
Are you interested to visit patients in the hospital or at home during the COVID-19 pandemic?	26 (5.8)	65 (14.4)	359 (79.8)	3 (3–3)	Never

**Table 4:** Demographic characteristics and median of Islamic attitude and practices among study population in KSA 2020.

Variables	Attitude		Practice	
	Median (IQR)	*p*-Value	Median (IQR)	*p*-Value
Age Group				
<20 years	40 (35–44)	0.070	21 (18–24)	≤0.001
20–30 years	41 (39–46)		22 (20–25)	
31–40 years	42 (38–46)		24 (21–26)	
41–50 years	42.5 (38–47)		24 (23–26)	
<50 years	38 (35–44)		24 (20–26)	
Gender				
Male	42 (36–46)	0.141	23 (20–25)	0.002
Female	42 (36–46)		24 (21–26)	
Nationality				
Citizens	42 (38–46.75)	0.525	23 (20–25)	0.001
Residents	42 (38–46)		24 (22–26)	
Education				
Less than university	42 (36.25–46)	0.075	21.5 (19–24)	≤0.001
University	41 (38–45)		23 (20–25)	
Post–graduate	42 (38–47)		24 (22–26)	
Marital status				
Not married	41 (38–45)	0.251	22 (20–25)	≤0.001
Married	42 (37.75–46)		24 (21–26)	
Divorced	49 (30–49)		24 (13–25)	
Occupation				
Employees	42 (38–47)	0.013	24 (20–26)	≤0.001
Freelance workers	39 (28–44)		23.5 (20–26.75)	
Students	41 (38–45)		22 (20–25)	
Housewives	46 (39.5–46)		23 (19.5–27)	
Job seekers	46 (38–49)		24 (18–25)	
Retired	35 (30–39)		20 (18–23)	

**Table 5:** Logisic regression predicting lower social and Islamic practice scores during COVID-19 pandemic in KSA.

Variables	OR (95% CI)	*p*-Value
Age Group		
<20 years	Reference	
20–30 years	0.64 (0.308–1.307)	0.217
31–40 years	0.38 (0.176–0.816)	0.013
41–50 years	0.23 (0.101–0.509)	>0.001
<50 years	0.38 (0.144–1.026)	0.050
Gender		
Female	Reference	
Male	1.65 (1.126–2.421)	0.01
Nationality		
Non-Saudi	Reference	
Saudi	1.57 (1.067–2.322)	0.022
Education		
Less than university	3.09 (1.721–5.563)	>0.001
University (Bachelor)	1.70 (1.122–2.585)	0.012
Post-graduate	Reference	
Attitude		
≥Median	Reference	
<Median	1.89 (1.309–2.753)	0.001

OR: Odd ratio; CI: Confidence interval.

**Table 6:** Rotated Component Matrix of Islamic attitudes among study population in KSA.

Items	Component	
	1	2
Suspending Umrah in Mecca to control the spread of the epidemic	**0.642**	−0.032
Preventing the elderly and people with chronic diseases to pray in the mosque	**0.692**	0.00
Preventing children under 15 years to pray in the mosque	**0.755**	0.039
2 m distancing between worshipers in mosque	**0.736**	−0.051
Wearing mask while praying in the mosque	**0.682**	−0.055
Shortening the time to stay in the mosque before and after prayer	**0.722**	0.021
Preventing the holding of Quran memorization sessions and Islamic instructional courses in mosques is necessary to the spread of the virus?	**0.688**	0.192
Prohibiting the hand-over of COVID-19 related-death to minimize the spread of the epidemic	0.045	**0.907**
Prohibiting the last look and kissing of COVID-19 related-death to minimize the spread of the infection	−0.049	**0.935**

Extracted factors are in bold.

**Table 7:** Association between demographic factors and two factors extracted by principal component analysis among study participants in KSA.

Variables	Worship		Dealing with Death	
	Median (IQR)	*p*-Value	Median (IQR)	*p*-Value
Age Group				
<20 years	30 (25–33)	0.016	6 (5–8)	≤0.001
20–30 years	31 (28–34)		7 (4–9)	
31–40 years	32 (26–35)		7 (5–9)	
41–50 years	30 (26–34)		9 (7–10)	
<50 years	28 (23–30)		8 (6–10)	
Gender				
Male	30 (25–34)	0.003	8 (6–10)	0.035
Female	32 (28–34)		7 (5–9)	
Nationality				
Residents	30 (27–34)	0.153	8 (6–10)	0.001
Citizen	31 (26–34)		7 (4–9)	
Education				
Less than university	30 (26–34)	0.269	7 (5–10)	0.007
University	31 (26–33)		7 (5–9)	
Post-graduate	31 (27–35)		8 (6–10)	
Marital status				
Not married	31 (27–34)	0.442	6 (4–9)	≤0.001
Married	30 (26–35)		8 (6–10)	
Divorced	35 (19–35)		10 (8–10)	
Occupation				
Employee	31 (26–34)	0.007	8 (6–10)	0.009
Freelance worker	28.5 (15–32.7)		6.5 (6–8.75)	
Student	31 (28–33)		6 (4–9)	
Housewife	35 (27.5–35)		6 (4–9)	
Job seekers	34 (27–35)		8 (7–9)	
Retired	23 (19–25)		8 (7–8)	

## Data Availability

The data presented in this study are available on request from the corresponding author. The data are not publicly available due to ethical issues.
